# Human T Cell Aging and the Impact of Persistent Viral Infections

**DOI:** 10.3389/fimmu.2013.00271

**Published:** 2013-09-13

**Authors:** T. Fülöp, A. Larbi, G. Pawelec

**Affiliations:** ^1^Geriatrics Division, Department of Medicine, Research Center on Aging, University of Sherbrooke, Sherbrooke, QC, Canada; ^2^Singapore Immunology Network, Biopolis, Agency for Science Technology and Research, Singapore, Singapore; ^3^Center for Medical Research, University of Tuebingen, Tuebingen, Germany

**Keywords:** aging, immunosenescence, T cells, CMV, chronic stimulation

## Abstract

Aging is associated with a dysregulation of the immune response, loosely termed “immunosenescence.” Each part of the immune system is influenced to some extent by the aging process. However, adaptive immunity seems more extensively affected and among all participating cells it is the T cells that are most altered. There is a large body of experimental work devoted to the investigation of age-associated differences in T cell phenotypes and functions in young and old individuals, but few longitudinal studies in humans actually delineating changes at the level of the individual. In most studies, the number and proportion of late-differentiated T cells, especially CD8+ T cells, is reported to be higher in the elderly than in the young. Limited longitudinal studies suggest that accumulation of these cells is a dynamic process and does indeed represent an age-associated change. Accumulations of such late-stage cells may contribute to the enhanced systemic pro-inflammatory milieu commonly seen in older people. We do not know exactly what causes these observed changes, but an understanding of the possible causes is now beginning to emerge. A favored hypothesis is that these events are at least partly due to the effects of the maintenance of essential immune surveillance against persistent viral infections, notably Cytomegalovirus (CMV), which may exhaust the immune system over time. It is still a matter of debate as to whether these changes are compensatory and beneficial or pathological and detrimental to the proper functioning of the immune system and whether they impact longevity. Here, we will review present knowledge of T cell changes with aging and their relation to chronic viral and possibly other persistent infections.

## Introduction

Aging affects all physiological systems, of which one of the most important interacting with and regulating many of the others is the immune system. This is composed of multiple cell types with specifically defined roles within a complex network of interactions. Immunologists and gerontologists have been interested in studying the effects of aging on the immune system for many years (1). Early work suggested that aging changed only adaptive immunity, but as analytical techniques became more refined, the innate arm was also found to be subject to age-related alterations. Nevertheless, T cells still seem to be most severely affected by aging (2). These changes may lead to clinical consequences such as the increased incidence of infections and most probably of cancers, cardiovascular diseases, and neurodegeneration in the elderly. However, despite a considerable amount of experimental work and knowledge we still do not know what causes these changes with age. Although changes in the innate immune response have been recently described (3–6) we will concentrate on changes in the human adaptive immune response. This review will describe changes in human T cell phenotypes, functions, and interactions as well as the possible causes of these changes, focusing on the effects of persistent viral infection.

## Immunosenescence

The aging of the immune system is a dynamic process which may at least partly reflect adaptation of the response to the evolving pathogen milieu (7, 8). Not all compartments of the immune response are aging in the same way, at the same speed or the same direction (9, 10). There was an assumption that all parts and functions of the immune system were decreasing with age, but currently the realization that compensatory increases may be developing over time is gaining ground. Even previously identified “senescent” T cells considered as anergic, have been shown to be able to maintain functions important for mounting effective immune responses (11, 12). Based on these findings, the question is whether these changes are exclusively detrimental or can be viewed as an adaptation mechanism to the exposures experienced by the aging organism over the lifetime (8). Better knowledge of these changes in human aging will also help to design treatments aimed at restoring appropriate immunity by identifying which components to manipulate.

The changes occurring in the immune system with aging are collectively designated “immunosenescence.” This appellation describes all the changes that occur in all parts of the immune response with aging, but strictly should be limited only to those shown to be truly deleterious. Of all the age-associated changes identified, those actually known to be deleterious are certainly in the minority. At the same time, these changes are accompanied by development of a low grade inflammatory status in many elderly people (13). Such “inflammaging” is more clearly associated than cellular changes with syndromes of aging including sarcopenia, cardiovascular disease, neurodegeneration, and physical frailty. It would perhaps be more accurate to call the global phenomenon occurring in the immune system the “Immunopause,” like many changes in the aging endocrine system, with less overtly negative implications.

The most noticeable changes are observed in the adaptive immune system and especially in T cells. T cells are the backbone of the adaptive immune response. The results of these changes are relatively well documented in the elderly. They are likely to be associated with an increased susceptibility to infections but indirectly possibly also to cancers, autoimmune disorders and chronic inflammatory diseases. T cell status is monitored primarily by surface marker phenotyping identifying the distribution of differentiation stages which we extrapolate to predict functional consequences, some leading to the above-mentioned diseases associated with aging. Several lines of experimental evidence suggest that aging is associated with an increase in memory cells, quantitatively more so in the CD8+ compartment but also in the CD4+ compartment (7, 14). Concomitantly, there is a decrease of the naïve T cells probably mainly because of the involution of the thymus with age (15, 16) and the continuous consumption of existing immunological resources (17) by new and persistent infections.

How can we phenotypically classify T cells? There are several surface markers which can be used. The most important are the receptors CCR7, CD28, and CD27 and the phosphatase CD45, which play important roles in T cell activation, proliferation, cytokine production, and lymph node homing. *In fine*, the disappearance of these markers from the cell surface may denotes functional alterations in these T cell properties (7). The use of these markers of differentiation/exhaustion/memory inflation (18) enables us to identify T cell differentiation stages from recent thymic emigrant naïve T cells to late-stage TEMRA cells. These surface markers can be used to distinguish T cell populations the majority of which will be naïve (N: CCR7+, CD27+++, CD28+++, CD45RA+), central memory cells (CM: CCR7+, CD27++, CD28++, CD45RA−), effector memory cells (EM: CCR7−, CD27±, CD28±, CD45RA−), and terminally differentiated memory cells re-expressing CD45RA (TEMRA: CCR7−, CD27−, CD28−, CD45RA+). This is currently the most widely accepted phenotyping model for CD8+ T cells but can be applied to some extent to CD4+ T cells as well. The memory cells protect the host from subsequent infections by the same pathogens. They survive and turn-over homeostatically, driven by IL-7 or IL-15 (19). Because thymic involution beginning early in life severely curtails the egress of fresh supplies of naïve cells to the periphery, the diversity and integrity of the T cell repertoire must be preserved by the homeostatic maintenance of naïve and memory cells (20).

**Figure 1:**
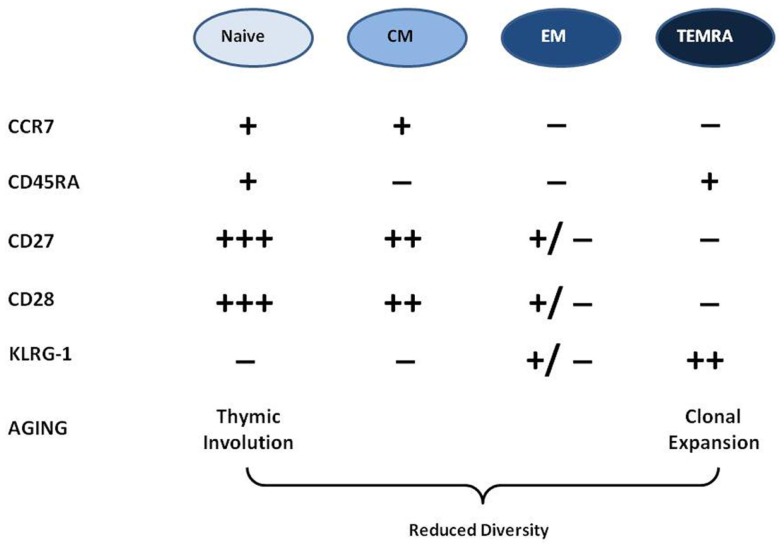
**Phenotypic characterization of T cells and their changes in aging**. There are four major differentiation stages of T cells characterized by the presence or absence of different surface markers in this popular model. With aging the naïve cell compartment is shrinking while the memory is expanding. There is also a loss of diversity with aging.

What does the picture look like in elderly individuals? The current paradigm is that the more we age (i) the more differentiated/memory T cells are accumulating because of the host’s immune exposure history (EM and TEMRA cells); (ii) the number of naïve T cells is decreasing because of thymic involution and exposure to specific antigen in the periphery; (iii) as there is a physical limit in the body to the number of circulating cells the accumulation of memory cells unbalances the naïve/memory ratio because clonally expanded memory cells are present in larger numbers than the naïve cells from which they were derived (1, 21) (Figure 1). There is a strong correlation between the chronological age and the frequency and absolute numbers of TEMRA CD8+ T cells in most human populations. Moreover, many of these TEMRA cells can be identified as dysfunctional in terms of cytokine response to stimulation and mediation of cytotoxic activity, as well as inability to proliferate. Hence, the accumulated population of late-stage memory cells includes those that might be senescent in the sense originally used by cell biologists, i.e., replicative senescence of fibroblasts, defined as permanent cell cycle arrest after a finite number of cell divisions (1, 22). The term “immunosenescence” is often confounded with this more restricted meaning of replicative senescence, especially because the lack of expression of the costimulatory receptor CD28 by late-stage CD8+ T cells is associated with essentially post-mitotic cells. Hence the notion that CD8+CD28− negative cells were senescent came to be common because of the role of CD28 in proliferation and functional commitment, in maintaining telomerase function, in p38 pathway activation, in modulating apoptosis and for adequate cell metabolism (23–25). By analogy to the replicatively senescent cells of Hayflick’s fibroblast cultures, those T cells which are not able to proliferate further were also considered as senescent. However, since these early observations were made, it has become evident that these differentiated T cells can under some circumstances regain proliferative capacity, such as after manipulation of KLRG1 (26, 27). The most likely explanation to account for the observed accumulation of late-stage memory cells in older humans is that while many are fully functional, this functionality no longer requires further clonal expansion of those antigen-specific cells. On the other hand, the accumulation of very large amounts of CD8+ TEMRA cells may result in overcrowding (shrinkage of the “immunological space” for other cells) because at least some of them are genuinely senescent.

## The Contribution of Longitudinal Studies

Swedish longitudinal studies were the first to investigate immune predictors of survival and death (28). Therefore, most of our findings concerning the accumulation of late-stage differentiated CD8+ T cells and whether this results in any negative consequences come from studies such as the OCTO and NONA longitudinal studies (29). These defined an “Immune Risk Profile” (IRP) as a cluster of parameters characterized by an inverted CD4/CD8 ratio due to an accumulation of CD8+CD28− T cells, associated with poor proliferative responses to T cell mitogens, but including low B cell counts and seropositivity for CMV. The IRP was defined a metric predictive of 2, 4 and 6 year mortality in people 85 years old at baseline. These findings largely contributed to our understanding of the cellular and molecular mechanisms underlying the decreased immune response with age (30). They characterized for the first time by TCR clonotype mapping the expansion of specific CD8+ T cell clones already described in mice and humans (31, 32). This decreased diversity of the total CD8 repertoire paralleled that of CD4 with age (33). Not only is there a decreased diversity, but these studies confirmed that the CD8 clones were strongly associated with CMV seropositivity (34), however in a much larger number of elderly and even older than in all other previous studies (30). It is now clear that persistent/latent CMV infection *per se* is associated with the accumulation of large populations of late-stage differentiated CD8+ T cells characterized by lack of expression of CD28 and acquired expression of the negative signaling receptor CD57, accompanied by decreased functionality (35). Moreover, in these Swedish studies it was also shown that the best predictor for survival was the inverted CD4/CD8 ratio (36) absent in “successful aging,” as epitomized by centenarians (37). The role of CMV in this respect was puzzling and to some extent remains so.

A seminal study by Simanek et al. (38) showed for the first time in a population of >10,000 adults that CMV positivity together with higher levels of the inflammatory marker CRP was associated with significantly poorer survival compared to those who did not have CMV seropositivity and/or CRP increased level. This remained the case in this NHANES cohort after correcting for multiple confounding factors. This suggests that CMV seropositivity is associated with poorer survival in people with increased background inflammation levels, borne out by such deaths being mostly cardiovascular. However, causation was not demonstrated and involvement of alterations in the immune system not shown (39). It also needs to be borne in mind that CMV infection *per se* has not been associated with increased background inflammation in all studies (40). Nonetheless, other studies also support the notion that CMV-seropositive elderly individuals have a higher propensity to suffer from other age-associated diseases with an inflammatory component, such as cardiovascular disease and cancer (14, 39, 41). Moreover, the IRP could rarely be found in other healthy populations or even in very ill elderly (42). So the question arises whether these changes are related to aging or to the chronic CMV infection or do they exist as an innocent bystander amplification?

## What Might then Cause these Changes in Adaptive Immunity with Age?

Recently a new paradigm emerged, mainly following these longitudinal studies, to explain the changes with age in T cell phenotypes and functions characterized by more and more differentiated CD4+ and CD8+ T cells: CMV seropositivity determines the increase of CD8+ TEMRA cells with a large proportion recognizing CMV antigens (43). It is of note that EBV-specific peripheral T cells are mainly effector memory phenotype, not so differentiated as for CMV. In parallel, EBV has not been strongly associated with immunosenescence and with mortality. Therefore, circumstantial evidence points to a unique effect of CMV on the accumulation of CD8+ TEMRA cells, presumably due to chronic antigenic stimulations by the persisting virus. However, this does not exclude the possibility that other viruses, or other completely different antigen sources, could have similar effects, for example, in the absence of CMV infection.

To better understand the eventual contribution of the different viruses we should clarify what persistent and chronic viral infections mean (44). Contrarily to acute viral infections, persistent infections have a longer duration mainly because the viral source is not cleared by the immune system and usually resides inside certain cell types (e.g., immune cells, neuronal cells, and epithelial cells). Persistent infections may involve stages of both silent and productive infection without rapidly killing or even producing excessive damage of the host cells. Varicella-zoster virus, measles virus, HIV-1, HHS-6, HHS-7, HSV-1, HSV-2, and human cytomegalovirus (CMV) are examples of viruses that cause typical persistent infections. Latent infection is characterized by the lack of demonstrable infectious virus between episodes of recurrent disease. A latent infection is a phase in certain viruses’ life cycles in which after initial infection, virus production ceases (HSVs, VZV). Chronic infection is characterized by the continued presence of infectious virus following the primary infection and may include chronic or recurrent disease. A chronic infection is a type of persistent infection that may be eventually cleared such as HBV, while latent infections such as EBV, CMV, and other herpes viruses persist for the lifetime of the host. Although persistent in nature their natural histories are completely different (44). It is not known why CMV has such a unique effect on immunity, or whether it remains latent in older people. Certainly, CMV reactivates periodically in adults, usually asymptomatically and therefore difficult to identify and study. Little is known about whether reactivation is more frequent on the elderly, and if so what the consequences are, if any.

**Figure 2:**
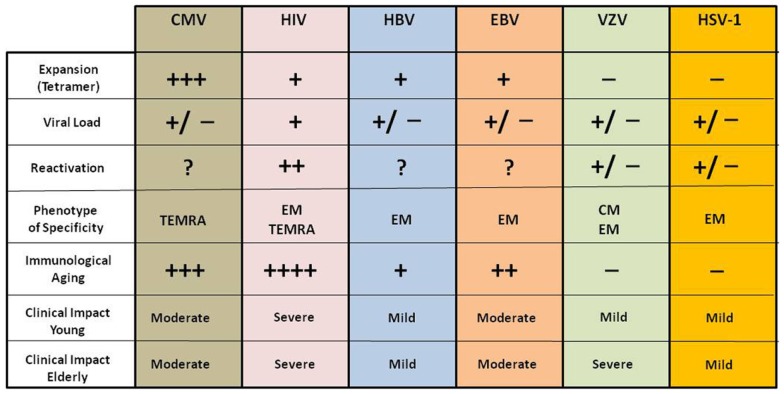
**Impact of different chronic viral infections on the immune system and clinical consequences in young and elderly subjects**. The immunological and clinical impact of these viral infections is different in young and elderly and even among elderly; however they present a persistent antigenic stimulation throughout life.

Thus CMV has evolved to avoid elimination by the hosts’ immune effector mechanisms and to persist mostly, presumably, in a non-replicative latent state (because CMV viremia is rare in healthy people). There is evidence to suggest that this latency is nevertheless a highly dynamic condition during which episodes of viral gene desilencing, which can be viewed as incomplete reactivations, cause intermittent antigenic activity that stimulates CD8 clonal expansion (8, 45, 46). All the other persistent virus have a different tissue specificity, reactivation inducers and amounts in the circulation as antigen resulting in a very different immune modulating effect compared to CMV (44, 47). We describe and summarize these differences in Figure 2.

### Chronic antigenic stimulation

The effect of chronic infection caused by CMV will be specifically discussed although various other chronic viral infections such as EBV, hepatitis B virus (HBV), human papilloma virus (HPV), or HIV (48) can also be considered as sources of chronic stress (Figure 2). It is well known that these viruses can reactivate when immunity is suppressed, suggesting that constant immune surveillance is required to prevent their reactivation (49, 50). They induce responses of virus-specific CD8+ T cells which can be detected long after the virus is controlled (51). Among these viruses HIV, inducing a distinct and well characterized viral disease at any age, was shown to be constantly replicating and causing an antigen-dependent clonal expansion of the memory T cells somewhat resembling what is found in the aging immune system (52). This led to a generalized concept of premature immunosenescence in HIV patients independent of their age (53). It is of note that basal viral load is constantly present in these individuals (44). Thus HBV (54), HIV (52), and CMV (55) are likely to drive the accumulation of memory T cells at any age, but not EBV and VZV due to their different life cycle and pathophysiology (47, 56, 57).

In this regard, HIV is very interesting and became important for the elderly since the success of antiretroviral therapy (ART). HIV was considered as a premature immunosenescence driver either for CD4+ or CD8+ T cells which showed certain characteristic surface markers and functions as found in elderly HIV uninfected people (53, 58). Many subjects suffering from AIDS now survive to become elderly and thus represent those with a different chronic viral infection from CMV. A persistent systemic inflammation characterizes the clinically latent or asymptomatic stage of the disease under good viral load control by ART (59, 60). Thus, there is continuous antigen stimulation, ongoing inflammatory process and CD4+ T cell loss. The chronological aging of HIV subjects is thus a good model to contrast with the known effects of CMV for discovering what is really due to a specific virus or to general virus-specific processes or to the aging process itself (53).

In addition to viruses, other stimulating agents may be present. This is perhaps the case for the continuous emergence of cancer antigens independent of the overt presence of clinical cancer (61). Indeed, many cancers such as advanced renal carcinoma, head and neck cancers, and melanomas are associated with the presence of late-differentiated CD8+ T cells (62, 63). Moreover, bacterial chronic/latent infections may also contribute; a good example might be Helicobacter pylori. Furthermore, one important source of continuous antigenic stimulation is the gut. It is conceivable that in aging the epithelial surface integrity is disrupted and becomes leaky to antigens coming directly from nutrients or from the gut microbiome, as is the case in HIV infection (64). These antigens can also drive T cells to late differentiation or exhaustion. Furthermore, CD28− CD8+ T cells are found in healthy elderly and in many disease states either in young or elderly subjects. This suggests that the origin of these cells is related to the disease more than the chronological age, and could be caused by chronic antigenic stimulation such as may occur in Alzheimer’s Disease (AD) or rheumatoid arthritis (65–67). In fact, we hypothesize that the increase of late-differentiated CD8+ and CD4+ T cells in AD is related to the constant presence of amyloid beta peptide. Therefore, the effects of these chronic antigenic stimulations may be quite different from that observed during chronic CMV infection. Thus, why CMV is so special?

### Chronic CMV stimulation

The fraction of the population infected with CMV depends on socio-economic status, with a prevalence is between 60 and 70% in industrialized countries, while in emerging countries it is almost 100%. Many studies were also performed in younger groups and children to confirm the persistence of this infection. It is of note that CMV may intermittently reactivate during the lifetime and must be maintained in a quiescent state to allow the remaining immune system to function. That is why the paradigm that constant antigenic stimulation by CMV induces immunosenescence was suggested. Nevertheless, it is of particular importance in this context that even young children infected with CMV have similar increased memory CD8+ profiles, decreased diversity in the naïve pool either and this led to the concept that CMV infection results in immune senescence at an earlier age (17, 68, 69). This would suggest that the so-called T cell senescence is not only age-dependent but CMV-dependent and what is seen in aging is the same as is observed in young or middle-aged subjects except that more time has passed since the initial infection. These findings underscore the general concept developed above that chronic antigenic stress is responsible for age-associated changes to the distribution of T cell differentiation phenotypes independent of chronological age, but increasing as age increases due to more extensive pathogen exposures throughout life.

The price paid by the adaptive immune system to maintain CMV in a latent phase is very high in terms of resource dedication (70, 71). In elderly subjects as many as 50% of CD8+ and 30% of CD4+ T cells can be CMV-specific (72) at the expense of the naïve and other more highly diversified memory T cells. The question is whether the remaining T cells are functional or not and this warrants further investigation. If they are not devoted to CMV their changes should be related to the aging process only, although “bystander” effects of the CMV-specific cells are conceivable too.

Current evidence suggests that CMV infection is a pre-eminent agent driving the differentiation of the CD8+ T cell compartment with aging (73–75) as has also been shown in mice (45, 76, 77). As mentioned above, most of these CMV-specific CD8+ T cells are of TEMRA phenotype with decreased CD28, CD27, CCR7, CD62L, and increased CD57 and re-expressed CD45RA surface markers indicating some putative functional alteration (52). These include decreased proliferative capacity (78, 79) but maintenance of markers of lineage commitment, memory, and cell–cell adhesion. However, even though some may be senescent, for example in terms of smaller proportions responding to specific antigen by producing IFN-γ, the absolute number of these cells is so greatly increased that the overall reactivity is enhanced, not decreased (80). This is also likely to contribute to the development of age-associated low grade inflammation and the consequent age-associated diseases (13, 81, 82). It is of note that this phenomenon is also seen in HIV-infected patients (53). Perhaps, as with the well-defined replicatively senescent cells these TEMRA CD8+ T cells may be largely pro-inflammatory producing a large amount of IL-6, TNFα, and IL-1β (83). However, more evidence has recently emerged that CD28-negative T cells are not necessarily (all) terminally differentiated or senescent because using specific costimuli such as 4-1BBL, OX40L, and cytokine supplementation with IL-2 or IL-15 revealed a retained proliferative capacity (11, 12). As they proliferate under IL-15 this implies that they are also sensitive to homeostatic control *in vivo* and cannot be considered as truly replicatively senescent (12, 84, 85). Furthermore, these TEMRA cells express high levels of Bcl-2 and are more apoptosis-resistant than naïve CD8+ T cells (34). This means that they will continue to accumulate over a longer period of time (i.e., during aging). The survival of these cells is energy-consuming. It is clear that in the competition for nutrients and factors to maintain these “functionally” different pro-inflammatory CD8+ T cells, they will largely outcompete the other T cell subpopulations. They have a survival advantage, with probably a very strong metabolic advantage too. Thus, their maintenance from a metabolic/energetic point of view is very costly and potentially harmful for the survival and function of other T cell subpopulations. This is further corroborated by the fact that CMV largely relies on the host’s mTOR pathway to maintain its viral reactivation potency from its latent form (86, 87). Thus, the presence of these metabolically very active CD8+ T cells may contribute to reactivation of CMV and thus to their further expansion, creating a vicious circle increasing with age. Consistent with this, recent evidence highlights and confirms the suppressive effect of mTOR toward CMV infection (87, 88). Thus, not only are these cells accumulating, but their maintenance in the system costs a lot of energy, thus depleting reserves, further impairing the composition and function of the remaining immune system.

In summary, apart from the robust Swedish longitudinal studies involving CMV infection/seropositivity in the so-called IRP, there is very little data to establish whether and how CMV positivity can further influence and shape the immune response in elderly subjects. There is no doubt that CMV is a contributory factor but certainly not the whole story (73, 74). Further longitudinal and functional studies are badly needed to establish the relationship between CMV and aging and mainly the true immunological and clinical effects of this relationship.

## What is the Significance of These Changes in the Perspective of Aging?

All these experimental data suggest that the dysregulation of the immune response or “immunopause” with aging may be due to a chronic antigen-driven process, especially but most likely not exclusively due to chronic CMV infection. The involvement of other factors as well as CMV must of course be recognized, if only because not all individuals are infected with CMV. Thus, there is a significant decrease in naïve T cells independent of CMV positivity even if CMV may further contribute to their decrease (55). CMV-negative individuals show less differentiated (CM and EM profile mainly) CD8+ T cells than CMV-positive elderly individuals (EM and TEMRA mainly) but this is just a question of proportion and both types are increased and have different functioning in elderly compared to young subjects. These CD8+ T cell phenotypic changes are also present in young CMV-positive individuals (66, 68), but we have no longitudinal data on the survival and T cell shifts in these young individuals throughout time. Additionally, the CD4+ compartment is also altered with aging (68, 72) but here CMV plays as much less prominent role than for CD8+ T cell differentiation.

## Clinical Consequences

The clinical consequences of the altered immune response with age are quite well-defined even if for most of them we have only circumstantial evidence. The increased infections, mainly new infections, indicate that naïve T cells’ functionality and/or diversity is altered also with aging and probably not only because of expanded CMV-specific T cells. It has been difficult to demonstrate that elderly have more active CMV infections than young subjects (89). Our own unpublished study showed that in elderly subjects suffering from pneumonia, we could not find acute CMV infection. This means that either the CMV-specific T cells are very efficient or that aging does not influence their functionality even if their numbers are increasing. It is of note that our actual clinical measure of CMV reactivation is far from being accurate.

An important clinical consequence of this increased CMV-specific CD8+ T cell clonally expanded pool with the concomitant decrease of the naïve T cell diversity is the decreased vaccine response in elderly (90). Thus, individuals having a large number of CD28− CD8+ T cells produce much less protective antibody following influenza vaccination than those having less of these cells (91). There were no studies on the CMV-specificity of these cells; nevertheless, regarding earlier studies it was speculated that CMV-induced CMV-specific CD8+ T cells decrease the response to influenza vaccine (92). However, in a different study, Saurwein-Teissl et al. (93) could not demonstrate any specificity of these CD8+ T cells for CMV, EBV or influenza viruses. The negative impact of CMV infection on the outcome of influenza vaccination in elderly people has been explicitly tested more recently and is seen in many studies [e.g., (94, 95)], but not all (96). However, the latter study in nursing home elderly only considered the antibody response to one of the three viral strains in that seasonal vaccine and found no difference in titers between CMV-infected and CMV-negative. As the WHO criteria for responsiveness specific reactions to two or more of the three strains, had this study examined the other two, a difference may have emerged. It is unclear why this was not reported.

Furthermore, CMV-infected individuals having expanded memory T cell populations have been shown to have a higher risk for coronary heart disease due to vascular inflammation (97, 98) and even for type 2 diabetes (99). Thus, CMV infection can be involved indirectly in the development of chronic inflammatory diseases which are increased in aging. Or vice versa the existence of these inflammatory diseases renders the elderly more susceptible to CMV reactivation. Nevertheless, as already mentioned, CMV positivity is associated with higher all cause of mortality than CMV seronegativity.

## Conclusion

**Figure 3:**
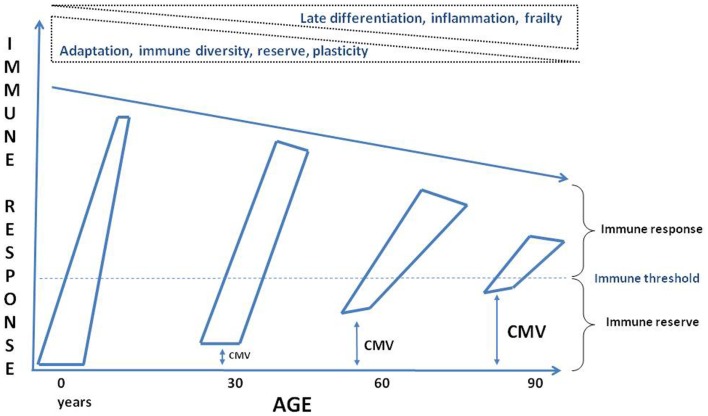
**Schematic conceptualization of the effects of aging on the immune system**. Through the progression of time the immune reserves are decreasing, the baseline is approaching to the threshold for inducing an efficient response, the immune response is less vigorous and maintained due to a complete reshaping of the immune cells by the immune history, such as persistent viral infection, putatively CMV. ↔: Represents possible CMV infection effects on the immune reserve and the threshold.

It is well known that aging is associated with increased susceptibility to many diseases due to dysregulated immune responses (Figure 3). However, we still do not know what the causes of this dysregulated immune response with aging really are. Here, it was argued that chronic antigenic stimulation, especially but not only due to CMV infection, plays a crucial role. However, dissecting the effects on immune alterations in elderly individuals with respect to age, low grade-inflammation, disease and CMV seropositivity remains a big challenge. We are currently approaching this challenge by assessing individual variations in responses to CMV, namely antibody titer, specificity, and neutralizing activity and determination of the specific CMV cell reservoirs (e.g., monocytes) rather than just “infected vs. not infected” (100). This approach appears to us more likely to yield informative data in populations where almost all subjects are infected with the virus, for instance elderly individuals even in industrialized countries and essentially everyone in developing countries. Furthermore, longitudinal studies are needed including young and elderly healthy individuals to dissect the effects of age vs. CMV infection. It is also warranted that comparable studies alone or in combination with other persistent viruses to be carried out to exactly determine their effects on the aging immune system. Finally, we should also consider that CMV infection is not uniquely a deleterious process but a constant trigger to maintain “alertness” in the aged immune system to be able to respond correctly to known antigens and overcoming the immunopause by the immunoadaptation triggered and maintained by persistent viral infection (Figure 3). Future experimental studies will help us to design efficient and intelligent interventions such as a vaccination or other means (gene silencing, antiviral agents, tissue-specific cell deletion, anti-inflammatory agents) to reinforce homeostasis and maintain a holistically adapted immune response to aging.

## Conflict of Interest Statement

The authors declare that the research was conducted in the absence of any commercial or financial relationships that could be construed as a potential conflict of interest.
